# Clinical severity according to the primary infection variant in patients with suspected SARS-CoV-2 reinfection in Korea

**DOI:** 10.4178/epih.e2023007

**Published:** 2022-12-21

**Authors:** Myung-Jae Hwang, Insob Hwang, Chungmin Park, Hanul Park, Taejong Son, Jong-Hun Kim

**Affiliations:** 1 Division of Infectious Disease Response, Gyeongbuk Regional Center for Disease Control and Prevention, Korea Disease Control and Prevention Agency, Daegu, Korea; 2 Department of Social and Preventive Medicine, Sungkyunkwan University School of Medicine, Suwon, Korea

**Keywords:** COVID-19, Reinfection, Vaccination, Disease severity, Omicron

## Abstract

**OBJECTIVES:**

We aimed to evaluate the severity of suspected severe acute respiratory syndrome coronavirus 2 (SARS-CoV-2) reinfection according to variants of concern in Gyeongsangbuk-do and Daegu, Korea.

**METHODS:**

The database of coronavirus disease 2019 (COVID-19) cases reported from epidemiological investigations through the integrated system operated by the Korea Disease Control and Prevention Agency, from January 20, 2020 to May 7, 2022 was combined with data from the Health Insurance Review and Assessment Service system. The severity odds ratio (S_OR_) in secondary infection episodes compared with primary infection was estimated using a generalized linear model with a binomial distribution.

**RESULTS:**

In all patients, the S_OR_ of SARS-CoV-2 reinfection was 0.89 (95% confidence interval [CI], 0.82 to 0.95), and the severity was lower than in the first infection. Patients who had been vaccinated within 91 days showed a more attenuated S_OR_ (0.85; 95% CI, 0.74 to 0.98). However, despite vaccination, in patients with both primary and secondary infections caused by the Omicron variant, the severity was reduced to a lesser extent than in patients primarily infected with other variants.

**CONCLUSIONS:**

We could make efforts to relieve the severity of COVID-19 in vulnerable populations, in which death is more likely, by recommending booster vaccinations in case of a resurgence.

## INTRODUCTION

The ongoing pandemic of severe acute respiratory syndrome coronavirus 2 (SARS-CoV-2) is characterized by its efficient transmission [1]. Evidence from animal studies indicates that the same or different strains of SARS-CoV-2 can cause reinfection more than 21-28 days after the initial infection [2-4]. This suggests that humans are also at risk of being reinfected. According to the World Health Organization (WHO), the Alpha (B.1.1), Beta (B.1.351), Gamma (P.1), Delta (B.1.617.2), and Omicron (B.1.1.529) variants affect the retransmission, severity, diagnosis, treatment, prevention, and infection control of coronavirus disease 2019 (COVID-19) [5-8]. Previous studies have shown that, compared to other variants, Omicron poses a higher risk of reinfection [8,9]. Considering the practical effectiveness of vaccines, public concerns and questions persist regarding the severity of reinfection with new variants [8]. Moreover, the population at risk for reinfection has increased due to the possibility of COVID-19 resurgence in Korea. In this study, we aimed to estimate disease severity based on the clinical database of patients with suspected SARS-CoV-2 reinfection according to the primary variant.

## MATERIALS AND METHODS

### Study subjects

Our study was conducted using the national database of confirmed COVID-19 patients reported from epidemiological investigations through the integrated system, which is a National Notifiable Disease Surveillance System operated by the Korea Disease Control and Prevention Agency (KDCA), from January 20, 2020, to May 7, 2022 in Gyeongsangbuk-do and Daegu. According to the KDCA guidelines [10], individuals with suspected reinfection were defined as (1) those with redetected positive COVID-19 test results more than 90 days after the first infection with or without symptoms, and (2) those with redetected positive COVID-19 test results in 45-89 days after the first infection, with symptoms, exposure to a confirmed patient, or a history of overseas travel.

During the study period, the number of patients with suspected SARS-CoV-2 reinfection was 4,297, and 4,283 patients were reinfected during the period of Omicron predominance (Table 1). Data were available on severity at the time of primary and secondary confirmation of suspected reinfection for 1,212 patients, and 1,171 patients were included to evaluate the severity of reinfection with Omicron by age group (Table 2). Among them, 824 patients received at least the first dose of COVID-19 vaccination (Table 3).

### Definition of the epidemic period

During the study period, the KDCA conducted next-generation sequencing (NGS) analyses only if the cycle threshold value was less than 28 for samples for which a polymerase chain reaction test was obtained among epidemiologically related patients with SARS-CoV-2 infection to evaluate the timing of periods when a new variant predominated (including re-detection as a result of contact tracing). Predominance was defined as a detection rate exceeding 50.0% or more. As a result of NGS analysis, we defined the epidemic periods as involving wild-type and Alpha predominance (from January 2020 to June 2021), Delta predominance (from July to November 2021), and Omicron predominance (after December 2021) considering the diagnoses and analyses of NGS and the overall epidemic situation in Korea.

### Defining the severity of infections

Based on the Health Insurance Review and Assessment Service system, the severity of infection was classified into 5 stages (asymptomatic, mild, moderate, severe, and critical) according to the WHO COVID-19 clinical severity scale at the time of hospital admission or treatment after confirmation [11]. We categorized the stage of asymptomatic or mild cases as low severity and moderate, severe, and critical cases as high severity in this study. The severity odds ratio (S_OR_) was estimated as the odds ratio for high severity versus low severity (as reference) in the secondary infection episode compared to the primary episode.

### Statistical analysis

A generalized linear model (GLM) with a binomial distribution was used to estimate the S_OR_ for secondary infection episodes compared to primary episodes. We calculated the S_OR_ with adjustment for the predominant variant at the time of the primary infection, as well as sex, age, duration after the last vaccination dose, and comorbid diseases affecting the cardiovascular and endocrine systems. All statistical analyses were performed using R version 4.0.4 (R Foundation for Statistical Computing, Vienna, Austria) software, and the level of significance was set at p=0.05.

### Ethics statement

Ethical approval was obtained from the Institutional Review Board of Sungkyunkwan University (IRB No. 2022-07-002).

## RESULTS

Table 1 summarizes the demographic characteristics of the patients with suspected reinfection. In total, 4,297 patients were reinfected, and most reinfections were reconfirmed during the Omicron-predominant period. Among 4,283 patients reinfected during the Omicron dominance period, 1,933 (45.1%) of the initial infection variants were wild type and Alpha, 1,389 (32.4%) were Delta, and 961 (22.5%) were Omicron. 1,568 (36.6%) of them had never been vaccinated.

Table 2 shows the estimated S_OR_ of patients suspected to have SARS-CoV-2 reinfection in the Omicron-predominant period compared to the severity of the primary infection according to age group. In all patients, the S_OR_ of reinfection was 0.89 (95% confidence interval [CI], 0.82 to 0.95), showing reduced severity compared with their first infection (S_OR_ < 1). The severity was attenuated to a lesser extent in the second infection in patients aged ≥ 60 years (S_OR_, 0.89; 95% CI, 0.73 to 1.09) than in those aged < 60 years (S_OR_, 0.88; 95% CI, 0.80 to 0.97). However, the S_OR_ was significantly increased in cases aged ≥ 60 years who were suspected to be infected with Omicron both primarily and reinfected (S_OR_, 1.20; 95% CI, 1.01 to 1.44).

Table 3 presents the S_OR_ of patients with suspected reinfection according to the period after their last vaccination dose. Among those vaccinated group within 91 days, vaccination significantly reduced severity. The S_OR_ was 0.85 (95% CI, 0.74 to 0.98) and declined to a greater extent in those vaccinated within 91 days than in those for whom more than 91 days had elapsed after vaccination (S_OR_, 0.91; 95% CI, 0.81 to 1.03). However, despite the effect of vaccination, in patients with primary and secondary infections caused by Omicron, the level of severity of reinfection was attenuated to a lesser extent than in those who were primarily infected with other variants. In patients who had been vaccinated within 91 days, the S_OR_ in the group primarily infected with Omicron was 1.46 times higher than that in the group primarily infected with the wild-type and Alpha variants. Furthermore, in patients for whom more than 91 days had elapsed after vaccination, the S_OR_ significantly increased to 1.08 (95% CI, 1.00 to 1.18) among those in whom the primary and secondary infections were caused by Omicron.

## DISCUSSION

We observed that the severity of reinfection was alleviated in patients reinfected after primary infection with wild-type/Alpha, Delta or Omicron. The reduced severity pattern was evident even in vulnerable patients aged ≥ 60 years, but in patients with 2 episodes of Omicron infection, the severity was worsened in reinfection. When compared to the primary infection, patients with COVID-19 caused by Omicron exhibited reduced severity, especially for those who had been vaccinated within 91 days [12]. Evidence supports a large decrease in severity (comparing Omicron with Delta) and the risk of hospitalization or death [13,14]. However, these effects were weaker in patients with Omicron infection for both primary and reinfection. This suggests that the risk of reinfection is higher with the Omicron variant, which has been shown to have a marked ability to evade immunity from previous infections [9]. The severity of reinfection declined in patients in whom less than 91 days had elapsed after their last vaccination dose [15]. Based on these findings, consistent with previous studies, we suggest that vaccination provides protection, including a reduction of disease severity in the first few weeks after vaccination; however, this protection weakens over time in some individuals [16].

Currently, we are facing a resurgence of COVID-19 infections with new recombinant variants. Therefore, we need to respond preemptively to the emergence of new variants and make efforts to implement pharmacological and non-pharmacological control strategies to protect against reinfection in elderly and vulnerable populations with low immunity due to a prolonged period after their last vaccination.

Our study has some limitations. First, the statistical analysis was performed on severity classified into 2 values as most of the cases did not have available severity information. Therefore, it was difficult to evaluate the level of severity attenuation using the existing classification system. Second, there was a possibility of misclassifying the variants responsible for infections according to the variant predominance period. In our study, since the predominance periods were defined based on the detection rates from NGS analyses, cases of reinfection during a certain predominance period may not have actually been caused by the predominant variant. Hence, the calculated S_OR_ may have been under-estimated or overestimated during the transitional period of variant predominance. Finally, it is also possible that the severity assessment may have been affected by the expansion of at-home treatment nationwide (from September 25, 2021) during the COVID-19 epidemic in Korea. Therefore, it is necessary to evaluate the severity with consideration of this change in the patient management system. Despite these limitations, the strength of this study lies in its evaluation of severity at the individual level of patients with suspected SARS-CoV-2 reinfection. In elderly patients with reinfection, the severity was mitigated to a lesser extent during the secondary infection episode. Vaccination was also observed to affect severity in patients with COVID-19 reinfection.

Based on this study, we could make efforts to minimize community outbreaks of new SARS-CoV-2 variants and expand vaccination to eligible populations, including those with a history of SARS-CoV-2 infection, to attenuate the severity in case of a COVID-19 resurgence in Korea.

## Figures and Tables

**Table 1.** Demographic characteristics of suspected patients with SARS-CoV-2 reinfection by variant in Gyeongsangbuk-do and Daegu, Korea, January 20, 2020 to May 7, 2022

Variables		Suspected patients with SARS-CoV-2 reinfection			
		Wild-type and Alpha predominant	Delta predominant	Omicron predominant	p-value^1^
Total		1 (100)	13 (100)	4,283 (100)	0.273
Sex					0.463
	Male	0 (0.0)	6 (46.2)	2,005 (46.8)	
	Female	1 (100)	7 (53.8)	2,278 (53.2)	
Age (yr)					0.925
	0-9	0 (0.0)	1 (7.6)	583 (13.6)	
	10-19	0 (0.0)	2 (15.4)	771 (18.0)	
	20-29	1 (100)	4 (30.8)	775 (18.1)	
	30-39	0 (0.0)	1 (7.7)	471 (11.0)	
	40-49	0 (0.0)	3 (23.1)	509 (11.9)	
	50-59	0 (0.0)	1 (7.7)	422 (9.9)	
	60-69	0 (0.0)	1 (7.7)	403 (9.4)	
	70-79	0 (0.0)	0 (0.0)	189 (4.4)	
	≥80	0 (0.0)	0 (0.0)	160 (3.7)	
Underlying comorbidity^2^					0.583
	Cardiovascular disease	0 (0.0)	0 (0.0)	252 (5.9)	
	Respiratory diseases	0 (0.0)	1 (7.7)	35 (0.8)	
	Endocrine diseases	0 (0.0)	0 (0.0)	136 (3.2)	
	Digestive diseases	0 (0.0)	0 (0.0)	14 (0.3)	
	Urinary disorders	0 (0.0)	0 (0.0)	11 (0.3)	
	Nervous system diseases	0 (0.0)	0 (0.0)	86 (2.0)	
	Mental disorders	0 (0.0)	0 (0.0)	95 (2.2)	
	Neoplasms	0 (0.0)	0 (0.0)	29 (0.7)	
	Others	0 (0.0)	1 (7.7)	19 (0.4)	
	None	1 (100)	8 (61.5)	1,938 (45.2)	
	Unknown	0 (0.0)	3 (23.1)	1,692 (39.5)	
Severity					<0.001
	Asymptomatic	0 (0.0)	6 (46.1)	114 (2.7)	
	Mild	1 (100)	5 (38.5)	1,050 (24.5)	
	Moderate	0 (0.0)	2 (15.4)	27 (0.6)	
	Severe	0 (0.0)	0 (0.0)	6 (0.1)	
	Critical	0 (0.0)	0 (0.0)	1 (0.0)	
	Unknown	0 (0.0)	0 (0.0)	3,085 (72.0)	
Vaccination					<0.05
	Not vaccinated	1 (100)	13 (100)	1,568 (36.6)	
	At least one dose	0 (0.0)	0 (0.0)	62 (1.4)	
	Fully vaccinated	0 (0.0)	0 (0.0)	1,266 (29.6)	
	First booster dose	0 (0.0)	0 (0.0)	1,324 (30.9)	
	Second booster dose	0 (0.0)	0 (0.0)	63 (1.5)	
Primary infection variants					<0.05
	Wild type and Alpha	1 (100)	11 (84.6)	1,933 (45.1)	
	Delta	0 (0.0)	2 (15.4)	1,389 (32.4)	
	Omicron	0 (0.0)	0 (0.0)	961 (22.5)	
Duration from primary infection to reinfection, mean±SD (day)		420.0	274.7±194.6	340.0±253.3	0.618

Values are presented as number (%). SARS-COV-2, severe acute respiratory syndrome coronavirus 2; SD, standard deviation.

1 Using the chi-square test or the Fisher exact test.

2 Included those with multiple underlying comorbidities.

**Table 2.** Severity odds ratio^1^ of SARS-CoV-2 reinfections with Omicron according to the variant responsible for the primary infection by age group in suspected cases

Primary infection variants	All	Age (yr)	
		<60	≥60
Total	0.89 (0.82, 0.95)^***^	0.88 (0.80, 0.97)^*^	0.89 (0.73, 1.09)
Wild-type and Alpha	0.86 (0.75, 0.99)^*^	0.70 (0.57, 0.87)^***^	0.86 (0.75, 0.99)^*^
Delta	0.88 (0.80, 0.96)^***^	0.89 (0.80, 0.99)^*^	0.94 (0.85, 1.03)
Omicron	1.17 (0.94, 1.45)	1.11 (0.96, 1.28)	1.20 (1.01, 1.44)^***^

Values are presented as severity odds ratio (95% confidence interval). SARS-CoV-2, severe acute respiratory syndrome coronavirus 2.

1 The odds ratio was calculated by the severity of reinfection compared with that of the primary infection, adjusting for the variant responsible for the primary infection, sex, age, duration after the last vaccination dose, and comorbid diseases affecting the cardiovascular and endocrine systems.

* p<0.05,

*** p<0.001.

**Table 3.** Severity odds ratio^1^ of patients with suspected reinfection according to the period after their last vaccination dose

Primary infection variants	Duration after last vaccination dose (day)	
	<91	≥91
Total	0.85 (0.74, 0.98)^***^	0.91 (0.81, 1.03)
Wild-type and Alpha	0.61 (0.49, 0.75)^***^	0.90 (0.77, 1.05)
Delta	0.86 (0.75, 0.98)^***^	0.92 (0.71, 1.18)
Omicron	0.89 (0.79, 1.00)	1.08 (1.00, 1.18)^*^

Values are presented as severity odds ratio (95% confidence interval).

1 The odds ratio was calculated by the severity of reinfection compared with that of the primary infection, adjusting for the variant responsible for the primary infection, sex, age, duration after the last vaccination dose, and comorbid diseases affecting the cardiovascular and endocrine systems.

* p<0.05,

*** p<0.001.
